# Biochemical Distribution of Tau Protein in Synaptosomal Fraction of Transgenic Mice Expressing Human P301L Tau

**DOI:** 10.3389/fneur.2014.00026

**Published:** 2014-03-11

**Authors:** Naruhiko Sahara, Miyuki Murayama, Makoto Higuchi, Tetsuya Suhara, Akihiko Takashima

**Affiliations:** ^1^Molecular Imaging Center, National Institute of Radiological Sciences, Chiba, Japan; ^2^Laboratory for Alzheimer’s Disease, RIKEN Brain Science Institute, Wako, Japan; ^3^Department of Aging Neurobiology, National Center for Geriatrics and Gerontology, Obu, Japan

**Keywords:** P301L tau, transgenic mice, subcellular fractions, synaptosomal fraction, tau phosphorylation

## Abstract

Alzheimer’s disease is a progressive dementia that is characterized by a loss of recent memory. Evidence has accumulated to support the hypothesis that synapses are critical storage sites for memory. However, it is still uncertain whether tau protein is involved in associative memory storage and whether tau is distributed in mature brain synapses. To address this question, we examined the synaptosomal distribution of tau protein in both JNPL3 transgenic mice expressing human P301L tau and non-transgenic littermates. The JNPL3 mouse line is known as one of the mouse models of human tauopathy that develop motor and behavioral deficits with intracellular tau aggregates in the spinal cord and brainstem. The phenotype of disease progression is highly dependent on strain background. In this study, we confirmed that male JNPL3 transgenic mice with C57BL/6J strain background showed neither any sign of motor deficits nor accumulation of hyperphosphorylated tau in the sarkosyl-insoluble fraction until 18 months of age. Subcellular fractionation analysis showed that both mouse tau and human P301L tau were present in the synaptosomal fraction. Those tau proteins were less-phosphorylated than tau in the cytosolic fraction. Human P301L tau was preferentially distributed in the synaptosomal fraction while mouse endogenous tau was more distributed in the cytosolic fraction. Interestingly, a human-specific tau band with phosphorylation at Ser199 and Ser396 was observed in the synaptosomal fraction of JNPL3 mice. This tau was not identical to either tau species in cytosolic fraction or a prominent hyperphosphorylated 64 kDa tau species that was altered to tau pathology. These results suggest that exogenous human P301L tau induces synaptosomal distribution of tau protein with a certain phosphorylation. Regulating the synaptosomal tau level might be a potential target for a therapeutic intervention directed at preventing neurodegeneration.

## Introduction

Neurofibrillary tangles (NFTs) and neuronal loss are commonly observed in neurological disorders, including Alzheimer’s disease (AD) and other tauopathies (1–3). Brain regions showing dysfunction overlap those displaying NFTs and neuronal loss (4), suggesting that mechanisms of NFT formation and neuronal loss may underlie neuronal dysfunction of affected brain areas. In frontotemporal dementia with Parkinsonism linked to tau on chromosome 17 (FTDP-17-*tau*), tau gene mutations induce NFT formation and neuronal loss (5–8), suggesting that dysregulation of tau may be a cause of NFT formation and neuronal death. This notion is supported by some reports showing that overexpression of FTDP-17-*tau* mutant tau induces NFT formation, neuronal loss, and behavioral abnormalities. In the mouse model rTg4510 overexpressing P301L mutant tau under the regulation of tetracycline, inhibition of mutant tau overexpression in the disease state blocked neuronal death and reversed memory impairment but still induced NFT formation (9), suggesting that NFTs themselves are not toxic, but the mechanism of neuronal death and memory impairment may underlie the process of NFT formation. Although the initial molecular event of tau pathogenesis remains unclear, the hyperphosphorylation of tau is strongly correlated with the severity of the pathology (10). The existence of hyperphosphorylated tau oligomers in human AD brain and transgenic mouse brains supports the idea of neurotoxic tau species (11–15).

Recently, several groups reported the mislocalization of hyperphosphorylated tau into dendritic spines (16–20). Interacting with Fyn kinase, tau contributes to NMDA stabilization (17, 21). Although a novel function of tau in post-synaptic regions was observed, evidence of hyperphosphorylated tau in dendritic spines still requires conclusive confirmation. On the other hand, it is well known that tau is involved in axonal transport stabilization and promotion of microtubule polymerization, and it participates in the transport of vesicles and organelles from axons to synaptic terminals (22). It was also reported that tau overexpression affects axonal transport by obstructing kinesin movement on microtubules (23–26). Since axon was labeled with Tau1 antibody, which recognizes non-phosphorylated tau at Ser199 (27), axonal tau seems to be de-phosphorylated. Therefore, it is important to clarify the status of tau phosphorylation in synaptic regions. In this study, we investigated the biochemical properties of synaptosomal tau extracted from transgenic mice expressing human P301L mutant tau.

## Materials and Methods

### JNPL3 mice and littermates

Male hemizygous JNPL3 mice were obtained from Taconic Labs (Germantown, NY, USA) at 8 weeks of age. JNPL3 mice express 4R0N isoform of human P301L mutant tau and are characterized as developing NFT, as well as sarkosyl-insoluble tau in an age-dependent manner (28, 29). Transgenic (Tg) mice and non-Tg littermates were bred by mating hemizygous JNPL3 mice with C57BL/6J Jcl (Clea, Tokyo, Japan). The mice were genotyped for the tau transgene by PCR between exons 9 and 13 of human tau cDNA. They were housed under controlled conditions with a 12-h day/night cycle. The age range of both male JNPL3 (*n* = 8) and male non-Tg mice (*n* = 5) was 15.7–18.5 months. Procedures involving animals and their care were approved by the Animal Care and Use Committee of RIKEN.

### Tissue extraction and subcellular fractionation

Mice were euthanized by cervical dislocation to preserve the brain metabolic environment and prevent artifacts that could alter tau biochemical profiles. Brains were quick-frozen on dry ice and stored at −80°C. For sarkosyl extraction, the cerebral cortex containing the hippocampus of the left hemibrain was subsequently homogenized in five volumes of Tris–buffer saline (TBS) containing protease and phosphatase inhibitors [25 mM Tris/HCl, pH 7.4, 150 mM NaCl, 1 mM EDTA, 1 mM EGTA, 5 mM sodium pyrophosphate, 30 mM β-glycerophosphate, 30 mM sodium fluoride, and 1 mM phenylmethylsulfonyl fluoride (PMSF)]. The homogenates were centrifuged at 27,000 × *g* for 20 min at 4°C to obtain the supernatant and pellet fractions. Pellets were re-homogenized in five volumes of high salt/sucrose buffer (0.8 M NaCl, 10% sucrose, 10 mM Tris/HCl, pH 7.4, 1 mM EGTA, 1 mM PMSF) and centrifuged as above. The supernatants were collected and incubated with sarkosyl (Sigma, St. Louis, MO, USA; 1% final concentration) for 1 h at 37°C, followed by centrifugation at 150,000 × *g* for 1 h at 4°C to obtain salt and sarkosyl-soluble and sarkosyl-insoluble pellets. The pellets were re-suspended in TE buffer (10 mM Tris/HCl, pH 8.0, 1 mM EDTA) to a volume equivalent to wet weight of the original tissue. For subcellular fractionation, fractions were prepared as previously described (30). Briefly, the cerebral cortex containing the hippocampus of the right hemibrain was Dounce-homogenized with 15 strokes in 10 volumes of homogenization buffer [25 mM Tris/HCl, pH 7.4, 9% sucrose, 2 mM EDTA, 5 mM dithiothreitol, 5 mM 4-(2-aminoethyl)-benzenesulfonyl fluoride hydrochloride (AEBSF), 5 ng/ml Antipain, 2 ng/ml aprotinin, 5 ng/ml leupeptin, 5 ng/ml pepstatin A, 1 μM okadaic acid, 1 mM NaF, 1 mM Na_3_VO_4_]. Nuclei (P1) were removed by 5 min centrifugation at 1,000 × *g*. The supernatant was subjected to 12,500 × *g* centrifugation for 15 min to yield the crude synaptosomal fraction (P2). The supernatant was centrifuged for 1 h at 176,000 × *g*, resulting in cytosol (S3) and light membrane and Golgi (P3) fractions. Synaptosomal fraction P2 was lysed hypo-osmotically and spun for 20 min at 25,000 × *g* to obtain the synaptosomal membrane fraction LP1. The supernatant was centrifuged for 2 h at 176,000 × *g*, resulting in a synaptic vesicle-enriched fraction (LP2) and a supernatant (LS2).

### Antibodies

E1 (31), a polyclonal antibody specific to human tau (aa 19-33, unphosphorylated), was prepared in our laboratory. MS06, a polyclonal antibody specific to mouse tau, was raised against mouse tau polypeptide corresponding to amino acid residue 118–131 (SKDRTGNDEKKAKG). Tau5, pS199, pT231, and pS396 were purchased from Biosource International (Camarillo, CA, USA). Tau1 was from Chemicon (Temecula, CA, USA). Monoclonal antibodies to β-actin and β-tubulin were purchased from Sigma. Monoclonal antibodies to GAP-43, PSD-95, and synaptotagmin were purchased from BD Transduction Laboratories (San Jose, CA, USA). For western blotting, antibodies were used at the following dilutions in blocking solution: E1, 1:5,000; MS06, 1:2,000; Tau1, 1:5,000; Tau5, 1:2,000; pS199, 1:5,000; pT231, 1:2,000; pS396, 1:2,000; β-actin, 1:5,000; β-tubulin, 1:5,000; GAP-43, 1:2,000; PSD-95, 1:2,000; synaptotagmin, 1:2,000.

### Western blotting

Fractionated tissue extracts were dissolved in sample buffer containing β-mercaptoethanol (0.01%). The samples were separated by gel electrophoresis on 10 or 5–20% gradient SDS-PAGE gels (Wako Pure Chemical Industries, Osaka, Japan), and transferred to nitrocellulose membranes (Schleicher & Schuell BioScience, Dassel, Germany). To estimate protein molecular weights, molecular size markers (Precision Plus Protein™ Standards, Bio-Rad Laboratories, Hercules, CA, USA; MagicMarker™ XP Western Protein Standard, Life Technologies, Carlsbad, CA, USA) were loaded on each gel. After blocking with a blocking solution containing 5% non-fat milk, 0.1% goat serum, and 0.1% Tween-20 in PBS, the membranes were incubated with various antibodies, washed to remove excess antibodies, and then incubated with peroxidase-conjugated, goat anti-rabbit antibodies (1:5000, Jackson ImmunoResearch, West Grove, PA, USA) or anti-mouse IgG (1:5000, Jackson ImmunoResearch). Bound antibodies were detected using an enhanced chemiluminescence system, SuperSignal West Pico (Pierce Biotechnology, Rockford, IL, USA). Quantitation and visual analysis of immunoreactivity were performed with a computer-linked LAS-3000 Bio-Imaging Analyzer System (Fujifilm, Tokyo, Japan) using the software program Image Gauge 3.0 (Fujifilm).

### Statistical analysis

Statistical analyses were conducted using PRISM4 (GraphPad Software Inc., La Jolla, CA, USA). Data were analyzed using the Friedman test or two-way ANOVA, unless otherwise noted.

## Results

### Examination of sarkosyl-insoluble tau in male JNPL3 mice

**Figure 1:**
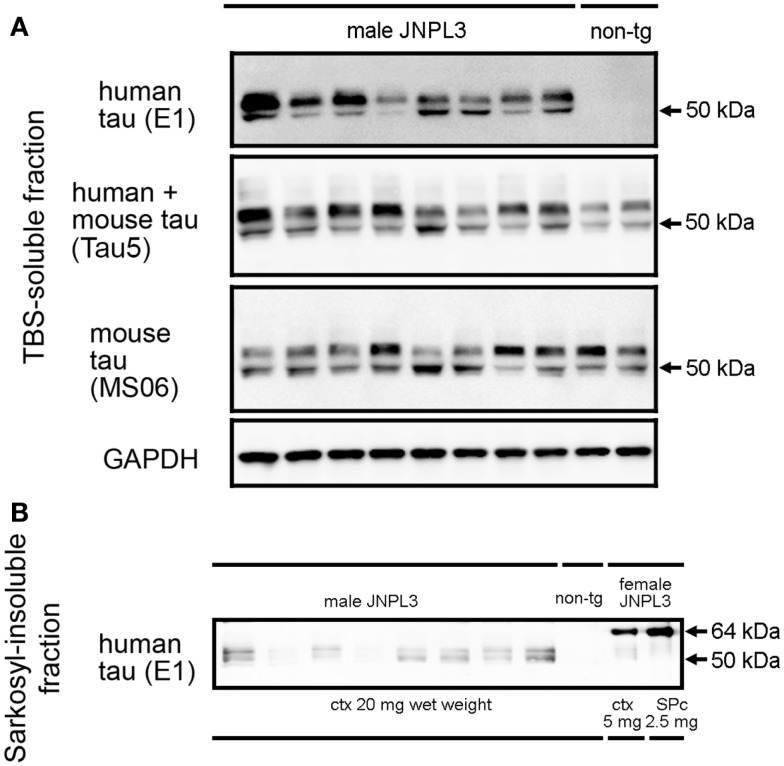
**Soluble and sarkosyl-insoluble tau in JNPL3 male mice**. **(A)** Western blots of TBS-soluble tau in mouse cerebral cortices. Equal volumes of TBS-soluble fraction derived from 0.2 mg wet weight of brain from eight male JNPL3 and two male non-tg mice were separated by SDS-PAGE, blotted, and then probed with E1, Tau5, MS06, and GAPDH antibodies. **(B)** Western blot of sarkosyl-insoluble fractions in mouse cerebral cortices. Samples derived from 20 mg wet weight from male JNPL3 and non-tg mice, 5 mg wet weight from female JNPL3 cortex (ctx), and 2.5 mg wet weight from female JNPL3 spinal cord (SPc) were separated by SDS-PAGE, blotted, and then probed with E1 antibody.

Hemizygous JNPL3 mice were reported to develop motor and behavioral deficits, initially presenting with hind-limb dysfunction starting at 6.5 months (28). These phenotypes were shown with a mixed C57BL/DBA2/SW strain background and mostly in female mice. Biochemically, subcortical regions contained more sarkosyl-insoluble tau than cortico-limbic regions, and male mice had less sarkosyl-insoluble tau than age-matched female mice (29). Moreover, a previous study showed that JNPL3 mice with C57BL/6J strain background significantly slowed the progression of tau pathology (32). In agreement with these reports, male hemizygous JNPL3 mice on an in-bred C57BL/6J strain at 15–18 months of age did not show any sign of motor deficits. To confirm whether male JNPL3 mice developed pathological tau aggregates in their brains, a sarkosyl-insoluble fraction was prepared using the sarkosyl extraction protocol as previously described (15, 29). Human and mouse tau proteins were detected in the TBS-soluble fraction using anti-human tau-specific antibody E1, anti-mouse tau-specific antibody MS06, and Tau5 antibody recognizing both tau (Figure 1A). We observed variable degrees of human tau protein among individual JNPL3 mice when normalized with GAPDH level (Figure 1A). In sarkosyl-insoluble fractions from male JNPL3 brains, the hyperphosphorylated tau migrating at 64 kDa was not found, although variable levels of 50 kDa tau bands were detected in this fraction (Figure 1B). The hyperphosphorylated 64 kDa tau was a marker of tau pathology in symptomatic JNPL3 and rTg4510 mice expressing the 4R0N isoform of human P301L mutant tau (15, 29). Because of undetectable levels of 64 kDa tau in male JNPL3 cerebral cortices (Figure 1B), these male JNPL3 cerebral cortices were considered to likely contain very few pathological tau inclusions.

### Subcellular distribution of tau protein in JNPL3 mice

**Figure 2:**
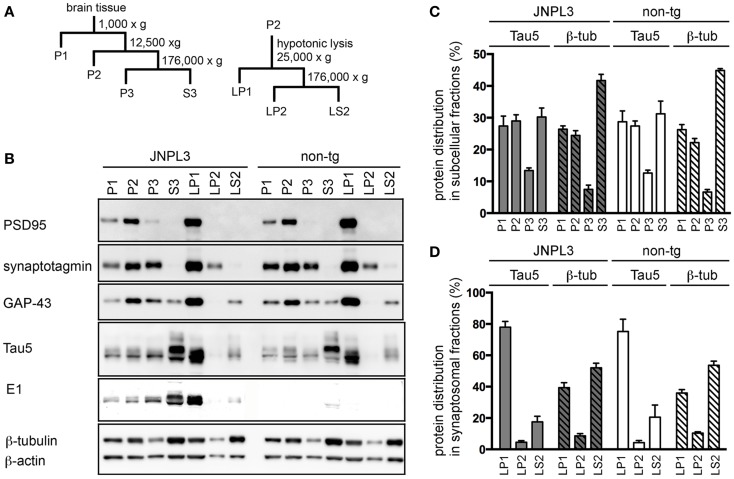
**Subcellular fractionation of mouse cerebral cortex**. **(A)** Schematic representation of the subcellular fractionation steps. P1, nuclear pellet and debris; P2, crude synaptosomal fraction; P3, light membranes; S3, cytosolic fraction; LP1, synaptosomal membrane fraction; LP2, synaptic vesicle-enriched fraction; LS2, soluble synaptosomal fraction. **(B)** Western blots of JNPL3 and non-tg male mouse cerebral cortex subcellular fractions. P1, P2, P3, or S3 fraction derived from 0.13 mg wet weight of tissue and LP1, LP2, or LS2 fraction derived from 0.5 mg wet weight of tissue were loaded on SDS-PAGE. Blots were probed with PSD-95, synaptotagmin, GAP-43, Tau5, E1, β-tubulin, and β-actin antibodies. **(C)** Proportions of protein levels in fractions (P1, P2, P3, and S3) of tau (Tau5) and β-tubulin (β-tub) from JNPL3 (*n* = 5) and non-tg (*n* = 5) mice are shown. Intensities of tau (49–65 kDa) and β-tubulin (50 kDa) were measured by Bio-Imaging Analyzer System. Ratios were indicated by percent of total (P1 + P2 + P3 + S3). Results are expressed as mean ± SEM. **(D)** Proportions of protein levels in synaptosomal fractions (LP1, LP2, and LS2) of tau (Tau5) and β-tubulin (β-tub) from JNPL3 (*n* = 5) and non-tg (*n* = 5) mice were indicated. Intensities of tau (49–65 kDa) and β-tubulin (50 kDa) were measured by Bio-Imaging Analyzer System. Ratios were indicated by percent of total (LP1 + LP2 + LS2). Results are expressed as mean ± SEM.

To identify tau protein in a synaptosomal fraction, we first performed subcellular fractionation and organelle enrichment by basic differential centrifugation protocol (30) (Figure 2A). As a result, pre-synaptic markers (GAP-43, synaptotagmin) and post-synaptic marker (PSD-95) were mostly distributed in the crude synaptosomal (P2) fraction (Figure 2B). Tau was recovered at equal ratios in the nuclear (P1), P2, and cytosolic (S3) fractions (Figure 2C). Distributions of tau, β-actin and β-tubulin in each fraction were similar in JNPL3 mice and non-Tg littermates (Figures 2B,C). Interestingly, the proportion of the tau level in S3 fraction was significantly lower than that of the β-tubulin level in S3 fraction (Figure 2C, tau and β-tubulin in S3 from JNPL3 were 30.2 and 41.7%, respectively, *p* < 0.05; tau and β-tubulin in S3 from non-tg were 31.2 and 44.9%, respectively, *p* < 0.01). This suggests that tau may have distinct functions rather than that of microtubule-association property. To further characterize the synaptosomal fraction, P2 fraction was lysed hypotonically and divided by differential centrifugation to obtain synaptosomal membrane (LP1), synaptic vesicle (LP2), and soluble synaptosomal (LS2) fractions (Figure 2A). GAP-43, synaptotagmin, and PSD-95 were mostly distributed in LP1 fraction (Figure 2B). Some of the synaptotagmin immunoreactivity was detected in LP2 as a synaptic vesicle protein (Figure 2B). Interestingly, abundant levels of both human and mouse tau were detected in LP1 but not in LP2 fraction (Figure 2B). Similar to the proportions of tau and β-tubulin S3 fraction, the proportion of the tau level in LP1 fraction was significantly higher than that of the β-tubulin level in LP1 fraction (Figure 2D, tau and β-tubulin in LP1 from JNPL3 were 77.9 and 39.4%, respectively, *p* < 0.001; tau and β-tubulin in LP1 from non-tg were 75.1 and 36.0%, respectively, *p* < 0.001). Again, tau may have a function that is not related to the microtubule-association. Furthermore, tau proteins in P1, P2, P3, and LP1 fractions had migrated faster than that in S3 fraction (Figure 2B, Tau5 and E1 blots), suggesting that posttranslational modification of those tau proteins was different from that of cytosolic tau protein.

### Tau in synaptosomal fraction

**Figure 3:**
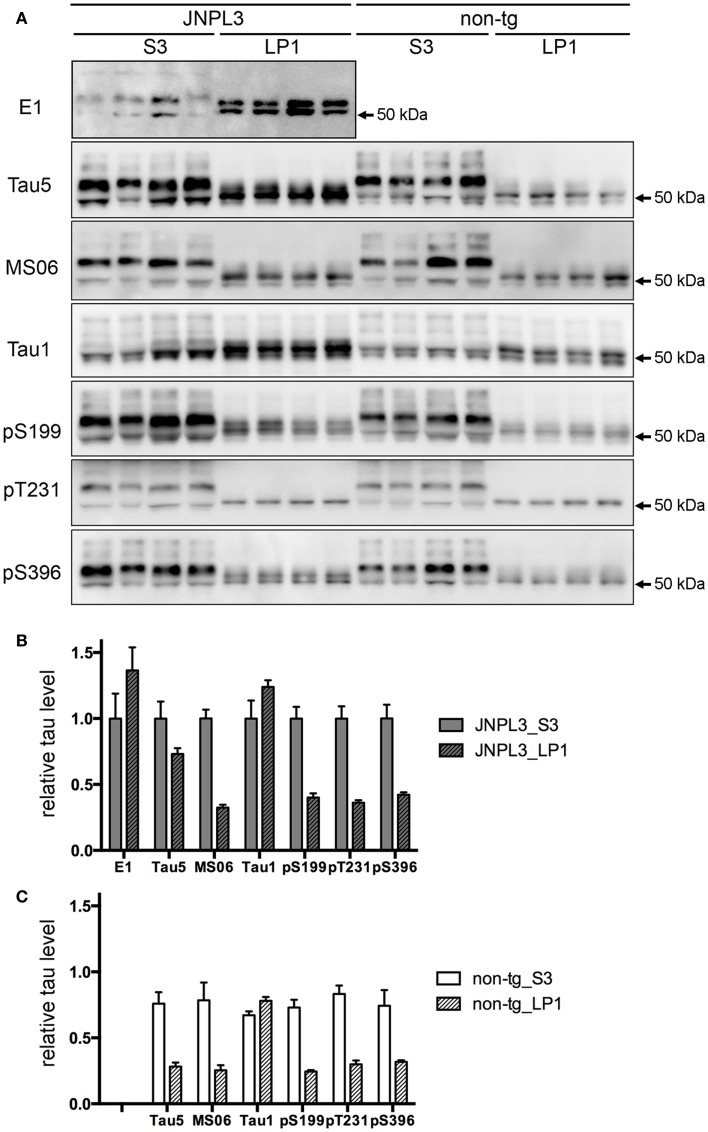
**Quantitative western blot analysis of tau protein**. **(A)** Tau band patterns in cytosolic (S3) and synaptosomal membrane (LP1) fractions from four JNPL3 and four non-tg mouse cerebral cortices. Equal volumes of fractions derived from 0.25 mg wet weight of brain were separated by SDS-PAGE, blotted, and then probed with Tau5, MS06, Tau1, pS199, pT231, and pS396 antibodies. **(B,C)** The relative ratio of tau protein between S3 and LP1 fractions from JNPL3 **(B)** and non-tg **(C)** mice was measured (*n* = 4 each). Results are expressed as mean ± SEM. The mean value of tau protein in S3 fraction from JNPL3 mice was normalized to one.

**Figure 4:**
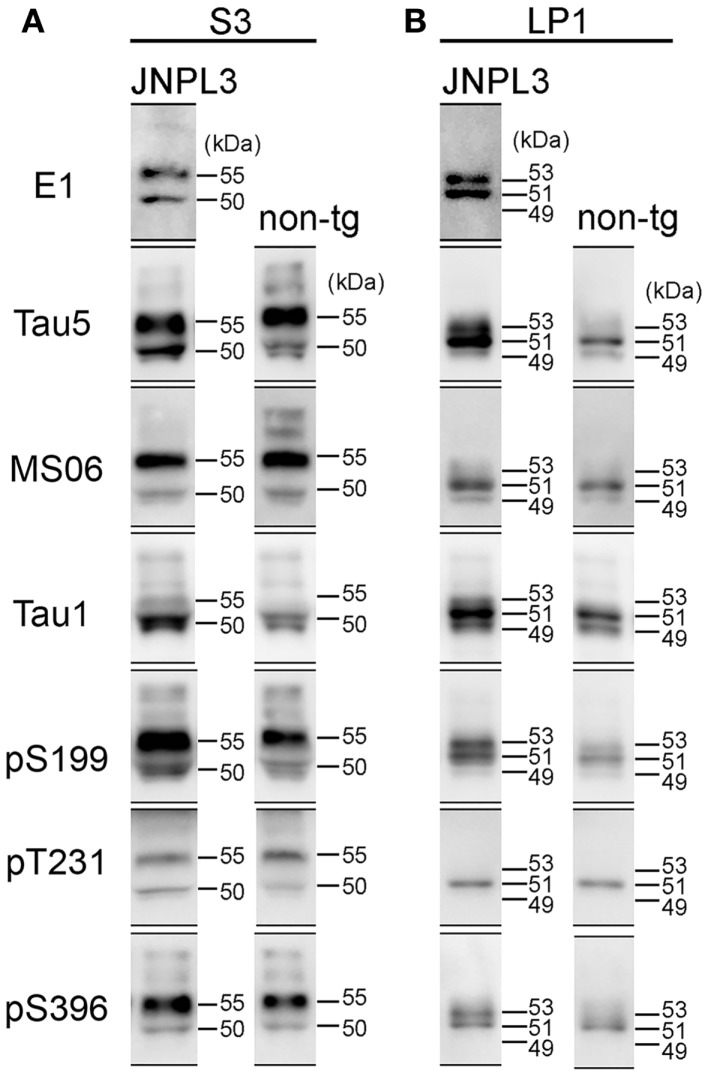
**Comparison of western blot profiles between JNPL3 and non-tg mice**. **(A)** Representative blots of S3 fraction stained by tau antibodies (E1, Tau5, MS06, Tau1, pS199, pT231, and pS396). Molecular weights were determined by reference standard markers. Two major bands were migrated to 50 and 55 kDa. Except Tau1 antibody, the 55 kDa band was more intensely stained than 50 kDa band. **(B)** Representative blots of LP1 fraction stained by tau antibodies (E1, Tau5, MS06, Tau1, pS199, pT231, and pS396). In JNPL3 mouse, two major bands appeared with slower migration (53 kDa) and faster migration (51 kDa) in E1, Tau5, pS199, and pS396 blots. Two bands appeared on 49 and 51 kDa in MS06 blot. Three bands (49, 51, and 53 kDa) appeared in Tau1 blot. Only 51 kDa band appeared in pT231 blot. In non-tg mouse, 49 and 51 kDa bands appeared in Tau5, MS06, and Tau1 blots. Three bands (49, 51, and 53 kDa) appeared in pS199 blot. Only 51 kDa band appeared in pT231 and pS396 blots.

To examine the effect of transgenic tau expression in the synaptosomal fraction, we next analyzed the protein property of tau in LP1 fraction. A panel of tau antibodies (E1, Tau5, MS06, Tau1, pS199, pT231, and pS396) confirmed the difference in band pattern between S3 and LP1 fractions (Figures 3A and 4A,B). When tau phosphorylations at the sites of Ser199, Thr231, and Ser396 were compared, those sites in LP1 were less-phosphorylated than in S3 fraction (Figures 3B,C). Tau1 antibody confirmed a higher level of de-phosphorylated tau in LP1 fraction than in S3 fraction of both JNPL3 and non-tg mice (Figures 3B,C). The ratio of tau phosphorylation between S3 and LP1 fractions of non-Tg mouse brain was similar to that of JNPL3 mouse brain [JNPL3, ratio of LP1/S3 = 1.24 (Tau1), 0.40 (pS199), 0.36 (pT231), 0.42 (pS396); non-tg, ratio of LP1/S3 = 1.16 (Tau1), 0.33 (pS199), 0.36 (pT231), 0.43 (pS396)] (Figures 3B,C). Total tau level in LP1 fraction from non-tg mice was also lower than that in S3 fraction [Tau5, LP1/S3 = 0.37] (Figure 3C). Although tau phosphorylations at the sites of Ser199, Thr231, and Ser396 in LP1 fraction of JNPL3 mice was clearly less than those in S3 fraction, mouse tau phosphorylation in LP1 fraction might be reduced at a few sites. Further analysis will be needed to determine differential phosphorylation sites in S3 and LP1 fractions of non-tg mice.

In JNPL3 mice, more human P301L mutant tau was detected in LP1 fraction than in S3 fraction (Figures 3A,B). In contrast, the total tau level including both human P301L tau and mouse tau was higher in S3 fraction than in LP1 fraction (Figures 3A,B). The higher level of human P301L tau was further confirmed by the lower level of mouse tau in LP1 fraction than in S3 fraction as detected by mouse tau-specific tau antibody MS06 (Figures 3A,B). These data indicate that the distribution of human P301L tau in the synaptosomal fraction is greater than in the cytosolic fraction while that of mouse tau is greater in the cytosolic fraction than in the synaptosomal fraction. It should be noted that Tau5 immunoreactivity in LP1 fraction of JNPL3 mice was more than twofold that of non-Tg mice (2.6-fold) while Tau5 immunoreactivity in S3 fraction of JNPL3 mice was less than twofold that of non-tg mice (1.3-fold). This further suggests the greater distribution of human P301L tau in LP1 fraction than in S3 fraction. Taken together, our biochemical analysis of the subcellular fraction revealed the existence of transgenic human P301L tau in the synaptosomal fraction with less-phosphorylation.

### Phosphorylated human P301L tau in synaptosomal fraction

Although the levels of tau phosphorylation in JNPL3 mice were similar to those in non-tg mice, we compared western blot profiles of tau protein between JNPL3 and non-tg mice. Molecular weights of major tau bands in S3 and LP1 fractions were estimated by protein standard markers (Figure 4). In S3 fraction of JNPL3 mice, two bands migrating to 50 and 55 kDa were labeled with tau antibodies E1, Tau5, MS06, pS199, pT231, and pS396 (Figure 4A). Tau1 antibody labeled the 50 kDa band but sparsely labeled the 55 kDa band (Figure 4A). Blot profiles of JNPL3 mice were not so different from those of non-tg mice (Figure 4A). JNPL3 mice expressed both human 4R0N tau isoform and mouse tau isoforms. In addition, it already is known that 4R0N isoform is most expressed in adult mouse brains (33). Therefore, the main components of these two bands might be human and mouse 4R0N tau isoforms, although the possibility of other mouse tau isoforms being induced by exogenous human tau cannot be excluded. In LP1 fraction, human tau appeared with 51 and 53 kDa bands (Figure 4B, E1 blot) whereas mouse tau appeared on 49 and 51 kDa bands (Figure 4B, MS06 blots). This phenomenon was also seen in blots of pS199 and pS396 antibodies (Figure 4B, 51 and 53 kDa bands in JNPL3, and 49 and 51 kDa bands in non-tg). The 53 kDa band was labeled with E1, Tau5, Tau1, pS199, and pS396 antibodies, but not with MS06 and pT231 antibodies. Because of slower mobility in SDS-PAGE, we expected the 53 kDa band to be more phosphorylated than the 51 kDa band. However, the same size of band was labeled with Tau1 antibody and not labeled with pT231 antibody, indicating that this 53 kDa band contained both phosphorylated tau at Ser199 and Ser396 and non-phosphorylated tau at Ser199 and Thr231. In addition, because the molecular size was quite different from the hyperphosphorylated 64 kDa tau (Figure 1B), the 53 kDa band was unlikely to be hyperphosphorylated. Regardless, identification of phosphorylation sites and tau isoforms will be necessary to determine the status of tau phosphorylation and expression in LP1 fraction.

## Discussion

Transgenic mouse models of human neurodegenerative diseases have been developed with the aim of reproducing the histopathological hallmarks of human diseases. For this purpose, neuropathology and biochemistry were commonly used for the definition of disease progress. Previous studies expressing transgenic tau under the control of neuronal promoters have successfully shown neuropathological hallmarks with the appearance of motor impairment (28, 34, 35). However, the phenotype did not completely match with AD because none of these tau transgenic lines had been comprehensively evaluated in terms of cognitive performance. Recently, rTg4510 mice were examined for the association of NFT formation with cognitive function, demonstrating that suppression of P301L tau expression reversed behavioral impairments although NFT formation continued (9). In rTg4510 mice and a neuronal cell culture model of tauopathy, the mislocalization of tau to dendritic spines was examined (16). Moreover, a novel function of tau altering Fyn kinase-mediated NMDA stabilization was reported (17, 21). However, it is still unclear whether abnormally hyperphosphorylated tau is accumulated in post-synaptic regions while having a function of NMDA stabilization. In the present study, instead of using a mouse model with extremely high expression of P301L tau, 15- to 18-month-old male JNPL3 mice were examined for their synaptosomal tau distribution. As previously reported (32), the hyperphosphorylated 64 kDa tau was barely detectable in the sarkosyl-insoluble fraction from male JNPL3 mice with a C57BL/6J strain background. Therefore, our biochemical analysis was unable to determine whether the hyperphosphorylated tau was distributed in synaptosomal fractions. However, we observed that less-phosphorylated tau was recovered in the synaptosomal fraction and that tau distribution was induced by exogenous human P301L tau expression. Interestingly, the sarkosyl-insoluble fraction extracted from male JNPL3 mice contained similar less-phosphorylated tau proteins (Figure 1B). It may be possible that transition from less-phosphorylation to hyperphosphorylation takes place in synaptic regions. Further study will be needed to confirm this possibility.

Among the cytoskeletal proteins showing polarized distribution, tau is abundantly localized in axons (27, 36–40). Therefore, it is reasonable to assume that the distribution of tau protein reaches the axon terminal. In fact, Fein et al. confirmed this by a flow cytometry method for detecting tau protein in synapses in AD brain (41). Here, we also showed significant amounts of tau protein in the synaptosomal fraction by using a subcellular fractionation method. Interestingly, the tau protein in this fraction was less-phosphorylated than that in the cytosolic fraction. Our quantitative analysis revealed that non-phosphorylated tau at Ser199 (detected by Tau1 antibody), as well as transgenic P301L mutant human tau, was distributed in the synaptosomal fraction. It was reported that the selective binding of tau to axonal microtubules was regulated by phosphorylation, as tau in axons was mostly stained by Tau1 antibody whereas tau in the cell body and dendrites was stained by AT8 antibody (42). Therefore, we speculated that P301L mutant human tau was functionally localized in the axon terminal with less-phosphorylation. In our synaptosome preparation with hypotonic lysis, tau was mostly recovered in the synaptosomal membrane fraction. As about 25% of cellular tau is reportedly located in membrane fractions (43), it is quite likely that tau exists in synapse in association with synaptosomal membrane components. At least, we know that tau can associate with various other proteins in addition to tubulin, including the SH3 domains of Src family tyrosine kinase (44). Because the synaptosomal membrane fraction contained more Tau1-positive tau than phosphorylated tau, most tau protein may localize in pre-synaptic regions. In contrast, localization of tau in dendritic spines was confirmed by the use of co-immunoprecipitation with PSD complex (17) and immunocytochemistry of EGFP-tagged tau expression in primary neuronal culture (16). In 12-month-old rTg4510 mice, a significant decrease in the synaptic protein level was biochemically demonstrated (20). These data strongly suggest that tau plays a critical role in the post-synaptic region in spite of the fact that the existence of hyperphosphorylated 64 kDa tau in dendritic spines of rTg4510 mice has not yet been conclusively proven. On the other hand, recent studies clarified that microtubule dynamics are essential for the regulation of spine morphology (45–47). Microtubule incursions into spines have been demonstrated in mature hippocampal cultures, suggesting that tau can be released at the plus-end of microtubules in spine. It was also reported that microtubule affinity-regulating kinase is essential for maintaining spine morphology (48). Therefore, the search for a transition mechanism of tau into the post-synaptic region is a project of major interest.

In conclusion, we observed that tau in the synaptosomal fraction was less-phosphorylated than that in the cytosolic fraction, and that synaptosomal tau distribution was induced by overexpression of human P301L tau in a transgenic mouse model of tauopathy. Although our data cannot determine whether the detected tau is of pre-synaptic or post-synaptic origin, further investigations will allow us to elucidate the critical role of tau in synapse. Regulating the synaptosomal tau level might be a target for therapeutic interventions to protect the formation of toxic tau species inducing synaptic dysfunction.

## Conflict of Interest Statement

The authors declare that the research was conducted in the absence of any commercial or financial relationships that could be construed as a potential conflict of interest.
